# The effect of movement therapy with progressive muscle relaxation on the depression rate of patients admitted to the psychiatric ward of Moradi Rafsanjan Educational and Medical Center in 2021

**DOI:** 10.25122/jml-2021-0436

**Published:** 2023-01

**Authors:** Ahmad Reza Sayadi, Ali Khodadadi, Ali Akbari, Zahra Abbasabadi

**Affiliations:** 1 Department of Psychiatric Nursing, School of Nursing and Midwifery, Rafsanjan University of Medical Sciences, Rafsanjan, Iran; 2 Social Determinants of Health Research Center, Rafsanjan University of Medical Sciences, Rafsanjan, Iran; 3 Department of Medical Surgical Nursing, School of Nursing and Midwifery, Rafsanjan University of Medical Sciences, Rafsanjan, Iran; 4 Department of Elderly, School of Nursing and Midwifery, Rafsanjan University of Medical Sciences, Rafsanjan, Iran

**Keywords:** movement therapy, progressive muscle relaxation, depression

## Abstract

Depression is the most prevalent psychiatric disorder and has received more attention due to its adverse outcomes, including suicide and a severe decrease in social and individual functioning. To this end, the present study examined the effect of movement therapy and progressive muscle relaxation on the depression rate in depressed patients. In the present interventional study, 60 patients diagnosed with major depression and hospitalized at Moradi Hospital's psychiatric ward in Rafsanjan in 2020, with an age of at least 20 years, were randomly divided into two groups: the intervention group and the control group. The subjects in the intervention group attended 30 sessions of 30–45 mins, with the researcher performing a movement therapy program followed by 15 to 20 minutes of progressive muscle relaxation. The Beck Depression Inventory was used to measure the degree of depression along with clinical pre-and post-intervention interviews. The mean depression scores were 37.26±7.70 and 36.93±8.166 for the participants in the intervention group and control group before the intervention, indicating no statistically significant intergroup difference (P=0.871). The mean depression scores after the intervention for the subjects in the intervention group and control group were 8.01±5.22 and 22.96±9.43, respectively. The results showed a statistically significant difference between the groups (P=0.001), with a greater decrease in depression scores in the intervention group compared to the control group. According to the present research, movement therapy and progressive muscle relaxation interventions effectively reduced depression in patients.

## INTRODUCTION

Mental disorders are a significant threat to overall health. Mood disorders, particularly depression, have become a major concern due to their negative impact on families, individuals, and society, including increased risk of divorce, suicide, increased illness burden, and decreased social functioning [1]. The World Health Organization predicts that by 2030, major depressive disorder will rank first in developed countries and second in developing countries in terms of disease burden [2].

According to the National Burden of Disease and Study in Iran, depression is the third health problem in the country. The prevalence of major depressive disorder over a lifetime is estimated at 5–17%. The American Psychiatric Association in 2013 reported a major depressive disorder occurrence rate of approximately 7% in one year. In Iran, it was estimated at 25%. This rate in Iran is 15 to 25% per year [3].

This disorder develops with no history of mania, mixed, or hypomania periods. The period of major depression should last at least two weeks. A patient diagnosed with major depression should have at least four signs on the list, including changes in weight and appetite, activity and sleep, lack of energy, feeling guilty, difficulty in thinking and making a decision, and persistent suicide and death thoughts [4]. Depression imposes significant economic, cultural, health, and social costs on patients and their families. These patients frequently occupy half of the beds in psychiatric hospitals due to recurrent episodes, leading to high costs for the mental health system [5]. Almost half of depressed patients do not respond completely to medication [6]. Depression results in reduced energy, disinterest, guilt, difficulty concentrating, anorexia, death and suicide thoughts, insomnia, oversleeping, and dysfunction [7]. Additionally, depression has been linked to an increased risk of developing conditions such as diabetes, stroke, and heart disease [8].

It is estimated that more than 50% of annual suicides are related to depression, and depressed patients are 20 times more likely to commit suicide than the rest of the community members [8]. Depression frequently manifests as a persistent and chronic condition that greatly impacts a person's mental well-being [9]. Its development is influenced by various factors and is characterized by diverse clinical symptoms and varying treatment responses, making its treatment difficult and incurring substantial costs for communities [10]. For the past four decades, two primary approaches have been used to treat depression: (1) medication for symptom relief and (2) cognitive-behavioral therapy. Antidepressants are the most commonly used treatment for depression, but relapse is common, with over half of those who recover from depression experiencing another episode within two years.

Medications are often used to reduce the likelihood of relapse in depression [9]. However, medications may not be the best option due to their side effects and expenses. Moreover, not everyone has access to counseling, highlighting the need for culturally-sensitive, evidence-based complementary therapies [11]. Recently, complementary medicine has been widely used in disease prevention and health promotion. The three most common methods of complementary medicine in the treatment of depression are movement therapy, progressive muscle relaxation, and painting therapy. These methods are easy and can be performed after a short training [12].

Physical activity and exercise, especially physical exercise-based interventions, have been suggested as an effective treatment and a good complement to medication [13]. Evidence suggests that while more severe depression is associated with lower levels of physical activity, increasing physical activity can reduce depression [14]. Progressive muscle relaxation was proposed by a physician named Jacobson in 1934 [15]. This method treats stress caused by chronic diseases and reduces anxiety, pain, and depression [7]. This is a kind of relaxation in which a person voluntarily increases blood flow and improves blood circulation by performing contractile movements and returning them to a relaxed and expanded state for 5 to 10 seconds. The muscles involved in this method include the right and left leg muscles, abdominal and lower back muscles, buttocks, chest and back muscles, right and left arm muscles, and neck and head muscles. The manner of performing this technique is so easy that it can be recorded on tape and given to the patient for practice at home [16]. Ghaffari *et al*. found such relaxation to reduce depression in patients with multiple sclerosis [7]. Klainin *et al*. also showed that progressive muscle relaxation effectively reduced depression and anxiety in older adults and reported that relaxation, music interventions and yoga had the strongest interventional effects on depression [17].

A review in 2015 showed that rhythmic movement therapy had no effect on depression and that further studies were needed to examine the effect of this therapy on all age groups [13].

Given the inconsistent research findings, the high prevalence of depression in society, its adverse psychological, social, and economic consequences, side effects, and medication and counseling costs, this research aimed to explore the impact of movement therapy with progressive muscle relaxation on depression among depressed patients hospitalized at the Moradi Hospital's psychiatric ward in Rafsanjan in 2020.

## MATERIAL AND METHODS

This study aimed to investigate the effectiveness of an experimental intervention on patients with major depression. The research was conducted on 60 patients with major depression admitted to Moradi Hospital in Rafsanjan. The sample size was calculated using the statistical formula:


$$n=2{\left(z_{1-\alpha /2}+z_{1-\beta }\right)}^{2}\sigma 2/d^{2}\left(\alpha =0.05,\;\beta =10\%,\;d=3,\;\sigma =3.35\right)$$

The minimum sample size required for the study was 26 participants, with an additional 30 participants in each group (intervention and control) to ensure a robust sample. The participants were all diagnosed with depression, admitted to the psychiatric ward of Moradi Hospital in Rafsanjan, and had a depression score of 21 or higher.

The inclusion criteria for the study were:

- Patients diagnosed with depression and admitted to the psychiatric ward;
- No history of musculoskeletal disorders;
- Ability to perform movement therapy and progressive muscle relaxation;
- A depression score of 21 or higher as per the standard measurement scales;
- No history of substance abuse or addiction.

Participants were selected using available sampling and random cluster sampling with the minimization method and were placed in two control and intervention groups. The purpose of minimization was to eliminate and control confounders (such as sex). Then, the Beck Depression Inventory was administered to the participants and psychological interviews to measure the level of depression. The researcher visited the psychiatric ward every evening and implemented a movement and progressive muscle relaxation program for the intervention group. The program lasted for 45 to 60 minutes and was performed by the participants.

The movement therapy program began with warm-up exercises, including walking and stretching for 5 to 10 minutes. Three stretching exercises were performed to warm up the muscles of the arms, legs, lower back, shoulder girdle, and neck. Then, the exercise, including 10 movements, gradually started from the hands to the legs. Each movement was repeated several times, and after performing every 2 or 3 movements, several deep breaths were taken in the standing position. At the end of the movements, stretching movements were performed for 5 to 10 minutes to cool the muscles again. Finally, muscle relaxation was performed for 15 to 20 minutes. Patients in both intervention and control groups received routine treatments.

The movement therapy and progressive muscle relaxation program were performed for 30 sessions. Upon the completion of the program, participants' level of depression was measured again using the Beck Depression Inventory and psychological interviews.

The data collected were codified and analyzed with SPSS software version 22. The Kolmogorov-Smirnov test, Fisher test, chi-square test, paired-samples t-test, independent samples t-test, as well as univariate analysis of covariance (ANCOVA) were used for data analysis. The significance level was 0.05 (P<0.05).

## RESULTS

The study findings revealed that the majority of patients in both the intervention and control groups were female. Most patients were under 40 years of age, with 11 patients in the intervention group and 13 in the control group. The mean age of the subjects in the intervention group was 47.06±11.45 and 40.66±11.45 in the control group. The chi-square test did not indicate any statistically significant difference between the groups in terms of age, and the two groups were considered homogenous in this regard (P=0.666). The majority of patients in both groups were married and had a middle school education. Most of the patients in both groups lived in cities. The participants in the groups were found to be similar in terms of the history of other physical and mental illnesses and duration of illness, which was mostly less than 3 years. Additionally, most patients in both groups had a history of taking medications.

The mean depression scores of the subjects in the groups were significantly different before and after the intervention (P<0.001). Furthermore, the mean difference for the subjects in the intervention group was 29.66 and that of the control group 13.96, indicating that the movement therapy and progressive muscle relaxation program significantly reduced the depression of patients in the intervention group compared with the control. Although the subjects in the control group reported a lower level of depression after the intervention than before, the differences in their depression scores were not significant (Table 1).

**Table 1:** Comparing the participants' mean depression scores in the two groups.

Groups	Pre-intervention scores	Post-intervention scores	Mean difference	Inter-group comparison
**Intervention**	37.26±7.70	8.01±5.22	29.66	t=18.240, df=29, P<0.001
**Control**	36.93±8.166	22.96±9.43	13.96	t=7.403, df=29, P<0.001
**Mean difference**	0.333	-14.966	-	-
**Inter-group comparison**	t=0.163, df=58, P<0.871	t=7.599, df=58, P<0.001	-	-

In order to compare the mean depression scores between the groups before and after the intervention, the independent samples t-test was used. The mean depression score for the subjects in the intervention group was 37.26±7.70, and 36.93±8.166 in the control group before the intervention. According to the independent samples t-test, no statistically significant difference was observed between the groups concerning their depression scores before the intervention (P=0.871). Furthermore, the mean depression score for the participants in the intervention group was 8.01±5.22, and 22.96±9.43 in the control group after the intervention. According to the independent samples t-test, a statistically significant difference was observed between the groups concerning their depression scores after the intervention (P=0.001), confirming a greater decrease in the scores of depression in the intervention group compared with the control (Table 1).

To evaluate the efficacy of the movement therapy program on depression in psychiatric inpatients, we conducted a univariate analysis of covariance (ANCOVA). Before conducting the analysis, assumptions were assessed by employing the Kolmogorov-Smirnov test to evaluate data normality and Levene's test to assess the homogeneity of error variance between groups. Since the F-value was not significant (P>0.05), the equality of the error variance for the two groups was confirmed, indicating no difference between them.

The results of the post-test showed a significant difference between the groups, with a significant F-value of 60.846 (p<0.00001) in their depression scores. The effect size of 0.516 indicates a substantial difference between the two groups, as shown in Table 2.

**Table 2:** The results of univariate ANCOVA for intergroup differences in depression.

Source	df	Mean square	F	Sig.	Effect size	Power
**Pretest**	1	196.211	3.518	0.066	-	-
**Group**	1	3393.233	60.846	0.00001	0.516	1
**Error**	57	55.678	-	-	-	-

Subsequently, the movement therapy program along with progressive muscle relaxation reduced the level of depression in the depressed patients admitted to the psychiatric ward.

## DISCUSSION

According to the study, no significant differences were observed between the control and intervention groups concerning demographic parameters, such as age, gender, marital status, place of residence, history of physical and mental illness, duration of illness, and history of medication, and thus the patients in the groups were homogeneous concerning the demographic features. The occurrence of major depressive disorder was twice as high in women compared to men almost all over the world and in all countries and cultures [8, 18].

The prevalence of depression was higher among women in this study. This disparity may be due to a higher prevalence of addiction among men in Rafsanjan, leading to their exclusion from the study based on the exclusion criteria. Consequently, the study population comprised a higher number of women. The highest frequency of depression was observed among patients under 40 years of age. The average age of onset of major depressive disorder is about forty years, and the age of onset in 50% of the patients in this study was 20 to 50 years [18]. Major depressive disorder is more common in individuals who have no intimate relationship or are divorced or separated [18, 19]. Most of the patients in this study were married. One possible reason is that most women in the study area are obedient to traditional cultural norms and consider divorce and separation a stigma. Moreover, most of the patients in this study had a middle school degree. Culturally, the lower the level of education, the higher the rate of depression [19].

This study also indicated a significant difference (P<0.001) in the mean depression score before the intervention (37.26±7.70) and after the intervention (8.01±5.22) in the intervention group. This finding confirmed the positive effect of movement therapy and progressive muscle relaxation on reducing depression in patients admitted to the psychiatric ward. This finding was consistent with the results of Salmon Katherine (2019) in the United States [20] and Gourgouvelis (2017) in Canada [20] and with studies conducted in Iran [13].

Exercise affects people's mental conditions by releasing endorphins and lowering the level of cortisol in the blood (that is secreted due to psychological distress). Endorphins relieve pain and thus create pleasant feelings when the individual is doing physical activity. The secretion of endorphins increases with exercise. Thus, more endorphins and serotonin are released in the body, changing people's moods and creating pleasant feelings [21].

One of the most important psychological benefits of physical exercises and activities is the social dimension. When doing exercise, the individual has to interact with others who may have many things in common, so the individual feels that he/she belongs to a group of people who are doing a useful activity, and thus he/she does not feel isolated and lonely. This socialization improves the individual's quality of life. Other benefits of exercise include improving self-esteem and self-confidence. When doing exercise, the individual feels that he/she is capable like other people and will eventually develop a higher level of self-confidence. Moreover, physical activity and exercise are effective in reducing depressive symptoms by creating a better body image [22]. However, according to Barrett *et al*., no significant relationship was observed between physical activity and the reduction of depression. These conflicting findings can be attributed to differences in participants' age and the number of movement therapy sessions in the two studies [23].

Consistent with the present study, Ramasamy *et al*. (2018) studied the impact of progressive muscle relaxation techniques in reducing anxiety and depression in patients with leprosy and showed a statistically significant difference in reducing patients' anxiety and depression before and after the application of this technique [23]. Furthermore, Khajehian *et al*. (2019) examined the efficiency of muscle relaxation exercises in reducing anxiety, stress, and depression in the elderly with type 2 diabetes. The results showed that patients' anxiety, stress, and depression reduced significantly after the intervention [24]. The body's response to muscle relaxation is a physiological phenomenon that activates the parasympathetic nervous system, and the person will feel relaxed. Progressive muscle relaxation produces natural chemicals in the body, which lead to the repair of cellular damage and the elimination of toxins. Moreover, enhancing mental resilience and boosting self-confidence can lead to improved performance, uncovering inner abilities, increasing intellectual prowess, and fostering creativity [25]. Naderi *et al*. (2017) examined the efficiency of muscle relaxation on anxiety, depression, and blood sugar in females with type I diabetes and showed that muscle relaxation does not affect depression in type I diabetic patients [26]. This finding was contrary to the results of the present study. One possible reason is that Naderi *et al*. (2017) examined only depressed patients with type I diabetes, while the present work focused on all patients with depression. In addition, the two studies used different interventions.

This study also indicated a statistically significant difference between the mean depression score before and after the intervention in the control subjects (P<0.001), showing routine treatment programs conducted for the patients in the control group also reduced their depression compared to the time before the intervention. However, this difference was much smaller than in the intervention group. Similarly, Gourgouvelis *et al*. (2018) found that exercise results in better clinical consequences in subjects who received medication and cognitive behavioral therapy for major depression [20]. In another study, Klainin-Yobas *et al*. (2015) examined the impact of relaxation intervention on anxiety and depression in older adults. They showed that older adults who received relaxation interventions in the control group also had a significant decrease in depression and anxiety. Still, this decrease was smaller compared to the patients in the intervention group [17]. Therefore, it can be argued that while drug therapy effectively treats depression and reduces depression scores after treatment, combining it with movement therapy and progressive muscle relaxation could lead to an even greater reduction in depression.

The mean depression scores for patients in the intervention group were 8.01±5.22, and 22.96±9.43 in the control group, after the intervention. The independent samples t-test indicated a statistically significant difference between the groups concerning their depression scores after the intervention (P=0.001), confirming a greater decrease in depression scores in the intervention group compared with the control group. Patients in this study were hospitalized and were all under medication. Drug therapy is the first line of treatment for psychiatric disorders. Therefore, after the intervention, there was a decrease in the average score of depression in the patients in the control group; however, this decrease was smaller compared to the subjects in the intervention group, as confirmed by Ghasemi *et al*. (2019) [27].

The results of the study suggest that physical exercise impacts human social development and influences various psychological and social traits. Self-esteem is one of the most important psychological and social characteristics of human beings and is affected by social interactions and physical activities. It is a feeling of self-worth, approval, emphasis, acceptance, and value that a person has towards themself. Self-esteem manifests in all daily human activities and is one of the main personality traits determining human behavioral characteristics [28]. Thus, it can be suggested that exercise increases self-esteem and reduces depression.

Accordingly, movement therapy and progressive muscle relaxation can be used along with medication to accelerate the improvement of depressive symptoms. Based on our findings, therapists can use these interventions in addition to drug therapy to treat depressed patients and achieve better and more reliable results. Progressive muscle relaxation leads to the production of natural chemicals that repair cell damage and eliminate toxins. It also strengthens mental strength and self-confidence, thereby increasing the efficiency and awakening of inner talents, creativity, and intellectual power. Furthermore, it creates a balance between the anterior and posterior hypothalamus. It ultimately reduces stress and anxiety, increasing the patient's concentration and memory and leading to higher physical and mental well-being [25]. This approach trains the mind to achieve peace by focusing on various body parts, creating a disconnection from the external environment and providing a sense of freedom [29–31].

## CONCLUSION

Our findings indicate that combining drug therapy with complementary therapies such as movement therapy and progressive muscle relaxation can be effective in treating depression. By incorporating an integrated approach, involving healthcare professionals like psychiatrists, nurses, and psychologists, patients, and their families may feel more confident in the treatment and treatment team.

Drug therapy is often considered the first line of treatment for mental illness in psychology. However, recent advancements in science and a shift towards incorporating modern medical techniques with traditional methods in developed countries have led to increased use of complementary medicine in treatment plans. These efforts aim to enhance the overall health and well-being of individuals in the community. Medical research has shown that combining pharmaceutical treatments and non-pharmaceutical therapies recommended in complementary medicine can effectively reduce psychological disorders. Previous studies have also supported the effectiveness of these therapies. Thus, the concurrent use of these treatments (medications plus complementary and alternative therapies) are more effective than each of these techniques alone. A limitation of this plan is that the outbreak of COVID-19 has prolonged the study period for observing adherence to health protocols.

## Personal thanks

The authors would like to appreciate the Vice-Chancellor for Research and Technology of Rafsanjan University of Medical Sciences and all the patients participating in this research, as well as the manager and staff of the psychiatric ward of Moradi Hospital.
